# Differences in Dietary Intake, Eating Occasion Timings and Eating Windows between Chronotypes in Adults Living with Type 2 Diabetes Mellitus

**DOI:** 10.3390/nu15183868

**Published:** 2023-09-05

**Authors:** Stanislava S. Katsarova, Emma Redman, Franciskos Arsenyadis, Emer M. Brady, Alex V. Rowlands, Charlotte L. Edwardson, Louise M. Goff, Kamlesh Khunti, Thomas Yates, Andrew P. Hall, Melanie J. Davies, Joseph Henson

**Affiliations:** 1 NIHR Leicester Biomedical Research Centre, Leicester General Hospital, College of Life Sciences, University of Leicester, Leicester LE5 4PW, UKjjh18@leicester.ac.uk (J.H.); 2 Diabetes Research Centre, University Hospitals of Leicester NHS Trust, Leicester LE5 4PW, UK; 3 Department of Cardiovascular Sciences, University of Leicester, Leicester LE1 7RH, UK; 4 Alliance for Research in Exercise, Nutrition and Activity (ARENA), Sansom Institute for Health Research, Division of Health Sciences, University of South Australia, Adelaide, SA 5001, Australia; 5 NIHR Applied Health Research Collaboration—East Midlands (NUHR ARC-EM), Leicester Diabetes Centre, Leicester LE5 4PW, UK; 6 Hanning Sleep Laboratory, Leicester General Hospital, Leicester LE5 4PW, UK; 7 Department of Health Sciences, University of Leicester, Leicester LE1 7RH, UK

**Keywords:** chronotype, chrono-nutrition, dietary intake, circadian rhythm, temporal distribution, eating window, eating occasions, meal timing, type 2 diabetes, obesity

## Abstract

Chronotype studies investigating dietary intake, eating occasions (EO) and eating windows (EW) are sparse in people with type 2 Diabetes mellitus (T2DM). This analysis reports data from the CODEC study. The Morningness-Eveningness questionnaire (MEQ) assessed chronotype preference. Diet diaries assessed dietary intake and temporal distribution. Regression analysis assessed whether dietary intake, EW, or EO differed by chronotype. 411 participants were included in this analysis. There were no differences in energy, macronutrient intake or EW between chronotypes. Compared to evening chronotypes, morning and intermediate chronotypes consumed 36.8 (95% CI: 11.1, 62.5) and 20.9 (95% CI: −2.1, 44.1) fewer milligrams of caffeine per day, respectively. Evening chronotypes woke up over an hour and a half later than morning (01:36 95% CI: 01:09, 02:03) and over half an hour later than intermediate chronotypes (00:45 95% CI: 00:21; 01:09. Evening chronotypes went to sleep over an hour and a half later than morning (01:48 95% CI: 01:23; 02:13) and an hour later than intermediate chronotypes (01:07 95% CI: 00:45; 01:30). Evening chronotypes’ EOs and last caffeine intake occurred later but relative to their sleep timings. Future research should investigate the impact of chronotype and dietary temporal distribution on glucose control to optimise T2DM interventions.

## 1. Introduction

The field of chronobiology has gained rapid interest over the past two decades particularly in relation to its potential influence on human health and disease [1]. The circadian clock is a complex system which controls the timing of physiological processes in the human body [2]. It is governed by the Suprachiasmatic Nucleus (SCN) which regulates peripheral clocks in organs and molecular clocks in cells. Disruptions in endogenous circadian rhythms resulting from environmental and behavioural factors can lead to desynchronisation between the SCN and peripheral clocks, also referred to as circadian misalignment [3]. This subsequently impacts metabolic and physiological processes, including nutrient absorption and energy expenditure [3,4]. Each organism develops its own circadian rhythm (~24 h) based on the stimuli in their environment (primarily light but also temperature and feeding) [2]. In humans, light stimulates wakefulness and feeding whereas darkness stimulates rest and fasting [3]. Due to this, when food intake is delayed and occurs during the resting phase, it can contribute to circadian misalignment [5].

An individual’s intrinsic circadian rhythm determines their preferred timing of sleep and activity which can be classified using chronotypes [6]. There are three main chronotypes—morning, intermediate (also referred to as “neither”) and evening [6]. Chrono-nutrition is a growing field which investigates the complex relationship between nutrition, circadian rhythms, metabolism and health [4]. Individuals with an evening chronotype tend to have delayed meal timing and greater energy intake later in the day [7]. They have also been found to engage in more unhealthy eating habits, have a higher body mass index (BMI) and higher risk of developing obesity and type 2 diabetes mellitus (T2DM) than other chronotypes [1,7,8]. Whilst it is well recognised that dietary factors have an imperative role in the prevention and management of T2DM [9,10,11], evidence on the impact of chronotype and dietary temporal distribution in people living with T2DM is lacking.

Within T2DM populations, evening chronotypes have been found to have poorer glycaemic control than other chronotypes [12,13]. The mechanisms for these observations are unclear due to limited studies but could be due to the delayed meal timing observed in evening chronotypes. Glucose tolerance is controlled by circadian rhythms and usually peaks during the daytime (when humans typically feed) and reduces during night time (when humans typically fast) [14]. Previous studies in healthy populations have observed that eating during the night, compared to the day, is associated with poorer glucose tolerance, reduced insulin sensitivity and misalignment between central and peripheral circadian rhythms [15,16].

Another aspect of dietary temporal distribution is the eating window (EW) which is defined as the duration of time between the first and last eating occasion (EO) in a 24-h period [17]. Time-restricted feeding (TRF) restricts the daily EW and extends the daily fasting window [18]. A shorter EW has been associated with improved glycaemic control and weight loss outcomes [15,16]. Two recent systematic reviews and meta-analysis found TRF to be superior in promoting weight loss and reduction in fasting blood glucose compared to non time-restricted interventions [19,20].

Early studies in people living with obesity have explored the inter-relationship between chronotypes and EW duration and shown chronotype-adjusted energy-restricted diets to be more effective in promoting weight loss than energy-restricted diets alone [21]. However, this has yet to be fully explored for people living with T2DM. There could be a benefit to prescribing a chronotype-adjusted dietary intervention to overweight/obese individuals with T2DM to elicit better weight loss outcomes which may positively impact glycaemic control. However, there is sparse evidence for the interaction between chronotype, dietary patterns and their temporal distribution in adults with T2DM [22,23,24]. Therefore, the aim of this secondary data analysis is to characterise the dietary intake, EW and timing of EOs by chronotype in a cohort of people living with T2DM.

## 2. Materials and Methods

Participants included in this analysis had data collected as part of the ongoing CODEC (“Chronotype of Patients with type 2 Diabetes and Effect on Glycaemic Control”) observational study (Clinical Trial Registry Number: NCT02973412) between 2016–2021. Ethical approval for this study was granted from the West Midlands—Black Country Research Ethics Committee (16/WM/0457). Full details of the study design and the cohort for the CODEC study have been described elsewhere [25]. Briefly, participants had established T2DM for more than 6 months, an HbA1c ≤ 86 mmol/mol (10%) and were aged between 18–75 years. Those living with Type 1 diabetes, a known sleep disorder (except obstructive sleep apnoea) or BMI over 45 kg/m^2^ were excluded. Eligible participants were recruited from both primary and secondary care settings from four sites across the Midlands, UK (Leicester, Nottingham, Derby, Lincoln). Written informed consent was received from all study participants. Participants working night shifts were excluded from this analysis (*n* = 1).

### 2.1. Chronotype

The Morningness-Eveningness self-assessment questionnaire (MEQ) was used to determine self-reported chronotype [26,27]. MEQ is a validated questionnaire comprising 19 items aimed at assessing individual differences in the degree of preference towards being active and alert during certain times of the day. The responses to the scale items indicate sleep and waking preferences and their subjective “peak” times. The chronotypes were categorised as either evening types (score of ≤52), intermediate type (53–64) or morning type (≥65) [27]. While chronotype is a continuous variable with a roughly bell-shaped distribution, for ease of analysis it is commonly divided into the above-mentioned three main types.

### 2.2. Dietary Assessment

In this sub-study, participants completed a three-day (*n* = 218) or four-day (*n* = 194) self-reported diet diary (including a minimum of two weekdays and one weekend day) to assess habitual dietary intake. Diet diaries not satisfying these criteria were excluded from the analysis. Participants were provided with written and verbal instructions on how to complete the diet diary and were asked to include details of all meals, drinks and supplements consumed as well as the timing of intake (temporal distribution). The collected diet diary data were input by trained members of the study team following standard operating procedures using the nutritional analysis software Nutritics (https://en-gb.nutritics.com/p/home). Estimates of intake for carbohydrate, protein, fat, caffeine (mg) and alcohol (units) intake were calculated by timed EO. EOs were assessed using the Gibney and Wolever (1997) definition: “an event which provides at least 210kJ (>50 kcal) with a separation in time from a preceding or following eating event of at least 15 min” [28]. Total daily (24 h) energy intake (kcal/day) and macronutrient intake (g/day) were also estimated.

To analyse the difference in temporal distribution of dietary intake between chronotypes, the average timing of participants’ first and last EOs was calculated by summing the time values of all valid days of data and dividing them by the number of days available (minimum 3 days). EWs were calculated by subtracting the timing of their last and first EOs. The duration of time between participants wake time and first EOs and the time between their last EO and sleep onset were calculated. The time interval between waking and first caffeine intake and last caffeine intake and sleep onset were also calculated. This was in order to assess the relative difference between EO timings, caffeine intake timings and wake/sleep onset across chronotypes.

### 2.3. Anthropometric, Demographic and Cardio-Metabolic Measures

Clinical characteristics were recorded by a trained member of the study team and included: age, sex (male/female), ethnicity (self-reported and categorised as (white European, South Asian, Black Caribbean or other)), duration of T2DM (years), number of T2DM medications, smoking status (current/ex/never), employment (employed/unemployed/retired/other) and body mass index (BMI; kg/m^2^), calculated to the nearest 0.1 kg/m^2^. HbA1c was quantified using the Bio-Rad Variant II HPLC system (Bio-Rad Clinical Diagnostics, Hemel Hempstead, UK).

### 2.4. Sleep Behaviours

Participants were also asked to wear an accelerometer (GENEActiv, ActivInsights Ltd., Kimbolton, UK) 24 h/day for 8 days to measure their habitual levels of sleep. The device was fitted on their non-dominant wrist during their data collection appointment and returned at the end of the assessment period. Alongside this, participants also completed a wake and sleep log for the days they wore the device. Accelerometer data were downloaded using GENEActiv PC Software version 3.2. Accelerometer data were processed using the R package GGIR version 1.8-1 (http://cran.r-project.org [29]) and the default GGIR sleep detection algorithm was applied to derive sleep duration and the mid-point of sleep [30]. Sleep logs were used to guide the algorithm to identify this sleep window. Participants’ data were excluded if their accelerometer files revealed post-calibration error > 0.01 g (10 mg), less than three days of valid wear (valid wear is defined as wear for over 16 h per day) or if wear data were not recorded for each 15 min period within the 24 h cycle. The following sleep characteristics were obtained: sleep duration (total accumulated sleep within the sleep window), discounting any wake time and daytime sleep, wake time, the time of sleep onset and the mid-point of sleep (to verify the self-reported MEQ data). The average of all valid days was used for all outcomes.

### 2.5. Index of Multiple Deprivation

The Index of Multiple Deprivation (IMD) was used to assess social deprivation. IMD scores are official assessments of relative compound social and material deprivation (accounting for education, employment, environment, health and income) in small areas in the United Kingdom. IMD scores are publicly available via the UK government website (https://imd-by-postcode.opendatacommunities.org/imd/2019) and are calculated using an individual’s postcode.

### 2.6. Statistical Analysis

Demographic, anthropometric, biochemical and accelerometer derived variables are presented as mean ± standard deviation (SD) for variables with a parametric distribution or median and interquartile range (IQR) for non-parametric variables. Categorical variables are presented as number and percentages. Multiple linear regression analyses assessed whether dietary intake, EW or timing of EOs and caffeine intake differed by self-reported chronotype. To look at independent effects, we adjusted for co-variates (age, sex, ethnicity, employment, duration of T2DM and IMD). A main effect of chronotype was followed by post-hoc contrasts using evening chronotype as the reference group. A sensitivity analysis also examined the impact of including sleep duration as a covariate in the fully adjusted model. All data were analysed using SPSS (version 24.0). A *p*-value of <0.05 was considered statistically significant for main effects. Results of the multiple linear regression are reported as mean (95% CI).

## 3. Results

At the time of analysis, 808 participants were enrolled in the CODEC study, of which 411 completed the diet diary and were included in the sub-analysis. Of these, all participants had anthropometric, demographic and diet data. Table 1 outlines the characteristics of all included participants, stratified by chronotype. Supplementary Table S1 outlines the participant characteristics of the wider CODEC cohort. Those included were broadly representative of the wider CODEC cohort. However, our sub-group cohort had fewer white Europeans (92.2% vs. 95.5%), older participants (65.2 vs. 62.9 years), lower HbA1c (6.9% vs. 7.2%) and BMI (30.6 kg/m^2^ vs. 31.4 kg/m^2^) than the wider CODEC population.

### 3.1. Chronotype

Chronotype was available for 406 participants; five participants did not have MEQ score data. Of the 406 participants included, 131 (32.3%) identified as morning, 195 (48%) as intermediate and 80 (19.7%) identified as evening chronotype. The participants MEQ scores were verified using midpoint of sleep data.

### 3.2. Dietary Intake

In total, 1367 days of diet data were available and reported in the analysis. Table 2 contains adjusted dietary intake data, stratified by chronotype. There were no significant differences in energy, carbohydrate, protein or fat intake across chronotypes. For unadjusted data, please see Supplementary Table S2.

### 3.3. Caffeine Intake and Timing

Caffeine intake differed significantly across the three chronotypes (*p* = 0.048) (see Table 2). Compared to evening chronotypes, morning and intermediate chronotypes consumed 36.8 (95% CI: 11.1, 62.5) and 20.9 (95% CI: −2.1, 44.1) fewer milligrams of caffeine per day, respectively (see Table 2). This would be equivalent to one less cup of coffee for the morning chronotypes and one less cup of black tea for the intermediate chronotypes [31]. There was a difference in the timing of last caffeine intake (*p* = 0.037) with evening chronotypes consuming caffeine over an hour later than morning chronotypes (01:06 hrs 95% CI: 00:34; 01:37) and half an hour later than intermediate chronotypes (00:33 hrs 95% CI: 00:05, 01:02). There was no difference in the timing of first caffeine intake across chronotypes.

There were no differences in the duration of time between waking and first caffeine intake between chronotypes. No differences were observed in the duration of time between last caffeine intake and sleep onset.

### 3.4. Wake Times and Sleep Onset

**Figure 1:**
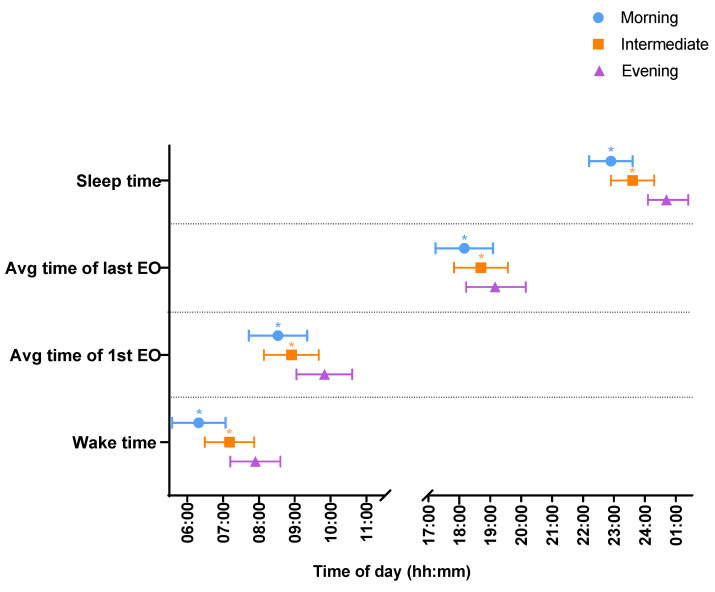
Timings of first and last EO, and sleep timings between morning (blue), intermediate (orange) and evening (purple) chronotypes. Time data are presented in the 24 h clock format. * *p* < 0.05 vs. evening chronotype.

Wake times differed across chronotype (*p* < 0.001) (see Figure 1 and Table 2), with evening types waking up an hour and a half later than morning chronotypes (01:36 95% CI: 01:09, 02:03) and over half an hour later than intermediate chronotypes (00:45 95% CI: 00:21; 01:09. Similarly, sleep onset differed across chronotype (*p* < 0.001) with evening types going to sleep over an hour and a half later than morning chronotypes (01:48 95% CI: 01:23; 02:13) and over an hour later than intermediate chronotypes (01:07 95% CI: 00:45; 01:30).

### 3.5. Eating Window and Timing of Eating Occasions

The timing of first and last EO differed across chronotypes (*p* < 0.001) (see Figure 1 and Table 2). Evening chronotypes had their first EO over an hour later than morning chronotypes (01:17 95% CI: 00:50; 01:45) and 55 min later than intermediate chronotypes (00:55 (95% CI: 00:30; 01:20). Similarly, evening chronotypes had their last EO over an hour later than morning chronotypes (01:06 95% CI: 00:34, 01:37) and half an hour later than intermediate chronotypes (00:33 95% CI: 00:05, 01:02).

There were no differences in the duration of time between waking and first EO between chronotypes. No differences were observed in the duration of time between last EO and sleep onset. There were no differences in EWs between chronotypes (see Table 2).

The interpretation for all results remained similar after further adjustment for sleep duration (see Supplementary Table S3).

## 4. Discussion

Our analysis of this cohort of adults living with T2DM found differences in the timings of EOs and caffeine intake by chronotype, with evening chronotypes consuming the most caffeine and eating later in the day. Importantly, these were relative to sleep timings which suggests that EWs shift in line with sleep patterns. This did not result in differences in energy and macronutrient intake or EW duration across morning, intermediate and evening chronotypes.

Our findings regarding dietary intake extend previous observations from healthy populations into a cohort living with T2DM. Regarding total daily energy intake, our findings mirror those that have found no difference across chronotypes [32,33,34]. However, our results are in contrast with findings from Mota et al., who found that evening chronotypes had significantly higher total daily energy intake compared to morning types [35]. Regarding macronutrient intake, most previous studies report no difference in macronutrient intake across chronotypes [32,36,37,38,39,40]. A small number of studies report conflicting results with some reporting higher carbohydrate intake in morning chronotypes [38,39,41] and others reporting higher carbohydrate intake in evening types [35,40]. Sato-Mito et al. and Mota et al. found that morning types had a higher protein intake compared to evening types [35,41]. Sato-Mito et al. and Maukonen et al. found that evening types had higher total fat intake compared to morning types [33,41]. The differences in the above-mentioned study findings compared to the current study could be attributed to different study populations (undergraduate students [36,41], adults (18–30 years [35], 18–50 years [32,38], 25–74 years [33])) living with or without obesity and using different dietary assessment methodologies (24 h recall vs. estimated diet diaries). Moreover, the studies were based in different countries with very different cultures and associated diets, customs and traditions (e.g., Brazil, Finland, Japan and the United States) which can affect dietary intake and habits.

Caffeine intakes in our cohort, although not excessive in comparison to recommended safe daily intake [42], were found to be higher in evening chronotypes compared to other chronotypes, which is in agreement with previous studies [43,44]. Only Bodur et al. quantified the amount of caffeine consumed in mg, and the average intake of caffeine of their study population was almost double that of our cohort’s [44]. Bodur et al. found that evening chronotypes also had poorer sleep quality and suggested that these individuals may be consuming more caffeine to compensate for the lack of sleep caused by waking up early to tend to social obligations [44]. However, in our cohort, a lower percentage of evening chronotype individuals were employed compared to those with morning chronotype preference which may have impacted our findings. Nonetheless, most of the published studies examining caffeine intake by chronotype recruited undergraduate students and used different methods to quantify the amount of caffeine consumed by participants which limits the generalisability of the mentioned findings. We also observed that evening chronotypes had their last intake of caffeine later than other chronotypes, although this was relative to their sleep onset. Penolazzi et al. also found that evening chronotypes have caffeine later in the day but did not find a difference in the amount of caffeine consumed [45]. However, they did not account for sleep timings.

We explored EW duration as TRF has been observed to positively impact glycaemic control and bodyweight control in people with T2DM and in people who have a shorter EW [18,46]. To our knowledge, there is only one previous study which examined EW duration and its relation to chronotype [47]; however, ours is the first study to investigate this in an adult population with T2DM. Gontijo et al. found no association between EW duration and chronotype in pregnant women but found that a longer EW was associated with better diet quality in the first trimester of pregnancy [47]. In our cohort, the cardio-metabolic profile, anthropometric measures and EW duration were similar across chronotypes.

The timing of EOs by chronotype reported here are in agreement with other studies exploring temporal feeding patterns across chronotypes and are consistent across different population groups [32,38,40,41,48]. These findings suggest that chronotype may be a predictor for the timing of EOs. The link between chronotype and EO timing preference could be an important area for future research since delayed food intake has been linked to circadian desynchronisation and metabolic disturbances [5]. To understand the relevance of meal timing and sleep behaviour, we looked at the relative difference between when individuals wake up and have their first EO and when they have their last EO and go to sleep. We found no difference in the relative duration of time between waking and first EO and last EO and sleep onset. This suggests that evening chronotypes are consuming their meals in line with their chronotype preference (therefore not in circadian misalignment). It is unclear whether delayed meal timing which is aligned with chronotype would still contribute to poorer glycaemic tolerance as suggested by previous studies which did not account for chronotype preference [14,15,16]. Therefore, further research is needed to examine the interplay between chronotype and meal timings on health outcomes.

Lending strength to our analysis is our well -phenotyped cohort which had anthropometric, diet and sleep data. However, there are a number of limitations. Due to the cross-sectional design of the CODEC study and derived data, we were not able to establish any causal relationships between the explored variables. In addition, the majority of our cohort consisted of people in retirement which could have influenced our findings. Although we determined chronotype using a validated questionnaire, across three pre-defined categories, it is possible that other statistical approaches (e.g., cluster analysis) may yield different results. We used self-reported diet diaries as our dietary data collection method which introduce limitations including the potential for misreporting of dietary intake and recall bias. To mitigate limitations, we used standardised diet diaries which collected information for both weekdays and weekend days to account for differences in dietary intake throughout the week. The diet diaries also contained detailed instructions and prompts to support accurate reporting, including images of food portion sizes. Despite this, our cohort’s dietary intake, particularly energy intake, is low compared to other studies in populations with T2DM [49], without T2DM (overweight/obese) [32,39] and with healthy BMI [34,36].

In conclusion, in our cohort of adults living with T2DM, we found no significant difference in dietary intake across chronotypes except for caffeine intake which was highest in evening chronotypes. We found that although there was a significant difference in the timing of EOs with evening chronotypes having delayed EOs, this was relative to their sleep timings. There was no difference in EW duration between chronotypes. Further research is needed to examine the association between dietary intake, temporal distribution and markers of cardiometabolic health. Future studies should also explore the impact of delayed meal timings on circadian rhythms and metabolic outcomes for people with T2DM particularly when accounting for chronotype preferences. This could help inform novel methods in glucose-lowering lifestyle interventions.

## Figures and Tables

**Table 1:** Participant characteristics for all participants and stratified by chronotype.

	All (*n* = 411)	Morning (*n* = 131) (32.3 %)	Intermediate (*n* = 195) (48%)	Evening (*n* = 80) (19.7%)
Demographic variables				
Age (years) (mean ± SD)	65.2 ± 7.6	64.8 ± 7.8	66.3 ± 6.8	62.9 ± 9.0
Sex (%female)	136 (33)	47 (35.6)	56 (28.7)	32 (40)
Current smokers (n%)	18 (4.4)	5 (3.8)	9 (4.6)	4 (5.0)
IMD	19,877.6 ± 8942	20,919.3 ± 8415.9	19,944.3 ± 8704.2	18,014.6 ± 10,229
Ethnicity (%)				
White European	380 (92.2)	126 (95.5)	178 (91.3)	72 (90)
South Asian	16 (3.9)	2 (1.5)	10 (5.1)	4 (5.0)
Black	7 (1.7)	2 (1.5)	4 (2.0)	1 (1.3)
Other	9 (2.2)	2 (1.5)	3 (1.5)	3 (3.8)
Employment (%)				
Employed	115 (27.8)	52 (39.4)	44 (22.6)	18 (22.5)
Retired	269 (65.2)	76 (57.6)	139 (71.3)	50 (62.5)
Unemployed	16 (3.8)	1 (0.8)	5 (2.6)	10 (12.5)
Other	12 (2.9)	3 (2.3)	7 (3.6)	2 (2.6)
Number of T2DM medications	1.3 ± 0.9	1.1 ± 0.9	1.4 ± 1.0	1.3 ± 0.9
Anthropometric variables				
BMI (kg/m^2^)	30.6 ± 5.2	30.6 ± 4.9	30.5 ± 5.1	30.9 ± 5.6
Cardio-metabolic variables				
HbA1c (%)	6.9 ± 1.3	6.7 ± 1.2	6.9 ± 1.2	7.0 ± 1.4
HbA1c (mmol/mol)	51.9 ± 14.2	49.7 ± 13.2	51.9 ± 13.2	53.0 ± 15.3
Duration of T2DM (years)	10.9 ± 7.4	9.7 ± 6.3	11.3 ± 7.6	11.9 ± 8.0

Data presented as median (interquartile range), number (percentage) or mean (±SD). The IMD score ranges from 1 (most deprived) to 32844 (least deprived). Time data are presented in the 24 h clock format. IMD—Index of multiple deprivation rank; BMI—Body mass index; T2DM—type 2 diabetes mellitus.

**Table 2:** Adjusted means for dietary variables, EW and EOs and sleep variables by chronotype with main effect.

Variable	Morning	Intermediate	Evening	Main Effect for Chronotype
Energy intake (kcal/d)	1530 (1246.0 to 1814.1)	1544.6 (1277.7 to 1811.5)	1504.5 (1233.2 to 1775.8)	0.428
Energy intake (kcal/kg)	22.7 (18.8 to 26.6)	22.3 (18.6 to 25.9)	21.3 (17.6 to 25.1)	0.257
Carbohydrates (g/day)	189.4 (155.9 to 223.0)	194.5 (163.3 to 226.3)	184.5 (152.4 to 216.5)	0.547
Fat (g/d)	57.9 (42.8 to 72.9)	56.2 (42.1 to 70.3)	57.4 (43.1 to 71.8)	0.507
Protein (g/d)	62.6 (48.5 to 76.7)	63.7 (50.4 to 77.0)	64.5 (51.0 to 77.9)	0.564
Caffeine (mg)	62.3 (16.5 to 108.1) *	78.1 (35.1 to 121.2)	99.1 (55.4 to 142.9)	**0.048**
Timing of first EO (hrs:min)	08:32 (07:47 to 09:21) *	08:54 (08:08 to 09:40) *	09:49 (09:03 to 10:36)	**<0.001**
Timing of last EO (hrs:min)	18:09 (17:14 to 19:05) *	18:42 (17:50 to 19:34) *	19:16 (18:22 to 20:09)	**<0.001**
EW (hrs:min)	09:35 (8:26 to 10:45)	09:46 (08:40 to 10:51)	09:21 (08:15 to 10:28)	0.595
Timing of first caffeine intake	10:31 (08:41 to 12:20)	11:13 (09:31 to 12:55)	11:19 (09:35 to 13:03)	0.297
Timing of last caffeine intake	12:33 (10:18 to 14:47) *	13:26 (11:20 to 15:33)	14:21 (12:13 to 16:29)	**0.037**
Time duration between waking and first EO (hrs:min)	02:11 (01:25 to 02:56)	01:58 (01:16 to 02:40)	01:53 (01:10 to 02:37)	0.570
Time duration between last EO and sleep onset (hrs:min)	04:50 (03:48 to 05:51)	04:53 (03:56 to 05:51)	05:21 (04:22 to 06:19)	0.309
Time duration between waking and first caffeine intake	04:10 (02:18 to 06:03)	04:05 (02:21 to 05:49)	03:22 (01:35 to 05:08)	0.284
Time duration between last caffeine intake and sleep onset	10:40 (08:24 to 12:55)	10:15 (08:09 to 12:21)	10:29 (08:21 to 12:38)	0.899
Wake time (hrs:min)	06:19 (05:35 to 07:04) *	07:11 (06:29 to 07:52) *	07:56 (07:13 to 08:38)	**<0.001**
Sleep onset (hrs:min)	22:58 (22:16 to 23:40) *	23:38 (22:59 to 00:18) *	00:46 (00:06 to 01:26)	**<0.001**

Linear regression models were adjusted for age, sex, ethnicity, employment, duration of T2DM and IMD. IMD—index of multiple deprivation rank; EO—eating occasion; EW—eating window. * *p* < 0.05 vs. evening chronotype.

## Data Availability

The data that support the findings of this study are available from the senior author [JH], upon reasonable request.

## Supplementary Materials

The following supporting information can be downloaded at: https://www.mdpi.com/article/10.3390/nu15183868/s1. Supplementary Table S1: Comparison of those include vs. remainder of CODEC cohort; Supplementary Table S2: Participant characteristics for all participants and stratified by chronotype (unadjusted data); Supplementary Table S3: Adjusted means for dietary variables, eating window and eating occasions and sleep variables by chronotype with main effect, including sleep duration as a co-variate.
