# Supplementary material for: Differences in Dietary Intake, Eating Occasion Timings and Eating Windows between Chronotypes in Adults Living with Type 2 Diabetes Mellitus
Source: Nutrients. 2023 Sep 5;15(18):3868. doi: 10.3390/nu15183868 (PMC10537296; doi:10.3390/nu15183868)
Supplement: Supplementary file 1 [file nutrients-15-03868-s001.zip › nutrients-2556299-supplementary.pdf]

Electronic Supplementary Material Table S1: Comparison of those include vs. remainder of CODEC cohort

	Included (n=411)	Remainder of CODEC cohort (n=562)	p-value for difference
**Demographic variables**			
Age (years) (mean ± SD)	65.2±7.6	62.9±8.7	<0.001
Sex (%female)	136 (33)	47 (35.6)	0.454
Current smokers	18 (4.4)	5 (3.8)	0.407
Index of multiple deprivation rank (IMD)	19877±8942	18384±9730	0.029
Ethnicity (%)			
White European	380 (92.2)	126 (95.5)	
South Asian	16 (3.9)	2 (1.5)	<0.001
Black	7 (1.7)	2 (1.5)	
Other	9 (2.2)	2 (1.5)	
Employment (%)			
Employed	115 (27.8)	52 (39.4)	0.001
Retired	269 (65.2)	76 (57.6)	
Un-employed	16 (3.8)	1 (0.8)	
Other	12 (2.9)	3 (2.3)	
Number of diabetes medications	1.3±0.9	1.5±1.0	<0.001
**Anthropometric variables**			
BMI (kg/m^2)	30.6±5.2	31.4±5.0	0.027
**Cardio-metabolic variables**			
HbA1c (%)	6.9±1.3	7.2±1.2	<0.001
HbA1c (mmol/mol)	51.9±14.2	55.1±13.2	
Duration of Type 2 diabetes (years)	10.9±7.4	11.0±7.4	0.835

Data presented as median (interquartile range), number (percentage) or mean (±SD). An independent t-test or chi-square tests were performed to assess differences in participant characteristics between the two cohorts.

Electronic supplementary table S2: Participant characteristics for all participants and stratified by chronotype (unadjusted data).

	All (n=411)	Morning (n=131) (32.3 %)	Intermediate (n=195) (48%)	Evening (n=80) (19.7%)
**Demographic variables**				
**Sleep variables**				
Wake time	07:20±01:53	06:36±01:46	07:22±01:07	08:33±02:49
Sleep onset (hrs:min)	23:25±01:36	22:45±01:30	23:33±01:02	24:19±02:20
Sleep duration (hrs:min)	07:41±01:10	07:37±01:07	07:48±01:08	07:25±01:25
Midpoint of sleep (hrs:min)	03:37±02:21	03:13±03:14	03:27±00:54	04:49±02:48
**Dietary Variables**				
Total Energy intake (kcal/d)	1623.5±463.0	1629.3±460.6	1627.7±471.2	1619.8±460.0
Energy intake per kg (kcal/kg)	18.8±6.38	19.0±6.7	18.9±6.3	18.6±6.0
Carbohydrates (g/day)	175.5±54.9	175.4±55.4	176.9±55.2	173.2±55.6
Carbohydrates (%)	43.8±7.7	43.7±7.8	44.0±7.8	43.2±7.2
Fat (g/d)	63.7±23.8	64.7±25.5	62.9±22.7	64.8±24.0
Fat (%)	34.6±6.7	34.7±6.5	34.4±6.8	35.3±7.0
Protein (g/d)	75.6±22.4	76.8±23.7	74.8±21.6	76.3±22.4
Protein (%)	19.2±4.5	19.3±4.4	19.0±4.3	19.5±5.2
Alcohol (units/d) (median with	1.0±12.2	1.5±9	1.0±9	1.0±7
Caffeine (mg/d) (median with	112.0±79.5	111.4±109.4	110.8±97.1	132.2±113.0
Eating window (hrs:min/d)	11:00±1:52	11:04±1:58	10:59±1:48	10:54±1:56
Timing of first eating occasion	08:38±1:27	08:11±1:33	08:39±1:18	09:21±1:25
Timing of last eating occasion	19:39±1:31	19:16±1:25	19:39±1:25	20:17±1:41
Timing of first caffeine intake	10:05±03:01	09:29±02:47	10:22±03:17	10:24±02:35
Timing of last caffeine intake	15:26±03:36	14:53±03:38	15:28±03:27	16:23±03:45

Duration of time between waking and first EO	01:34±01:18	01:49±01:37	01:30±01:05	01:20±01:11
Duration of time between last EO and sleep onset	03:51±01:39	03:36±01:30	03:52±01:37	04:18±01:58
Duration of time between waking and first caffeine	02:58±03:07	03:06±02:53	03:07±03:18	02:18±02:53
Duration of time between last caffeine intake	08:05±03:35	08:10±03:37	07:58±03:18	08:13±04:17

Data presented as median (interquartile range), number (percentage) or mean (±SD).

Electronic supplementary table S3: Adjusted means for dietary variables, eating window and eating occasions and sleep variables by chronotype with main effect, including sleep duration as a co-variate.

Variable	Morning	Intermediate	Evening	Main effect for chronotype
Energy intake (kcal/d)	1542.2 (1256.1 to 1828.4)	1554.8 (1288.5 to 1821.2)	1496.7 (1223.8 to 1769.5)	0.483
Energy intake (kcal/kg)	23.1 (19.1 to 27.2)	22.5 (18.8 to 26.3)	21.1 (17.3 to 25.0)	0.240
Carbohydrates (g/day)	194.3 (161.3 to 227.3)	196.5 (165.9 to 227.2)	181.6 (150 to 213.0)	0.299
Fat (g/d)	57.6 (42.3 to 72.8)	56.4 (42.2 to 70.6)	57.2 (42.6 to 71.7)	0.810
Protein (g/d)	60.0 (46.0 to 73.9)	63.7 (50.7 to 76.6)	64.7 (51.4 to 77.9)	0.459
Caffeine (mg)	55.0 (9.5 to 100.5)*	77.9 (35.6 to 120.3)	102.0 (58.6 to 145.4)	**0.008**
Timing of first EO (hrs:min)	08:35 (07:47 to 09:22)*	08:59 (08:14 to 09:43)*	10:01 (09:15 to 10:46)	**<0.001**
Timing of last EO (hrs:min)	18:11 (17:16 to 19:05)*	18:43 (17:52 to 19:34)*	19:27 (18:35 to 20:20)	**<0.001**
Eating window (hrs:min)	09:33 (8:23 to 10:42)	09:41 (08:36 to 10:46)	09:20 (08:14 to 10:27)	0.754
Timing of first caffeine intake	10:34 (08:44 to 12:24)	11:11 (09:29 to 12:54)	11:22 (09:38 to 13:07)	0.403
Timing of last caffeine intake	12:22 (10:09 to 14:35)*	13:29 (11:25 to 15:33)	14:22 (12:15 to 16:28)	**0.022**
Duration of time between waking and first EO (hrs:min)	02:04 (01:21 to 02:47)	01:54 (01:14 to 02:35)	01:43 (01:02 to 02:25)	0.475
Duration of time between last EO and sleep onset (hrs:min)	04:40 (03:43 to 05:37)	04:49 (03:56 to 05:42)	05:05 (04:10 to 05:59)	0.586
Duration of time between waking and first caffeine intake	04:01 (02:11 to 05:50)	03:57 (02:16 to 05:39)	03:06 (01:22 to 04:50)	0.148
Duration of time between last caffeine intake and sleep onset	10:31 (08:17 to 12:44)	10:08 (08:04 to 12:12)	10:14 (08:08 to 12:21)	0.912
Wake time (hrs:min)	06:31 (05:54 to 07:08)*	07:17 (06:42 to 07:51)*	08:14 (07:39 to 08:50)	**<0.001**
Sleep onset (hrs:min)	22:48 (22:11 to 23:25)*	23:33 (22:59 to 00:08)*	00:31 (23:56 to 01:06)	**<0.001**

Multiple linear regression models were adjusted for age, sex, ethnicity, employment, duration of T2DM, IMD and sleep duration.
